# Immunological fingerprint in coronavirus disease-19 convalescents with and without post-COVID syndrome

**DOI:** 10.3389/fmed.2023.1129288

**Published:** 2023-04-24

**Authors:** Max Augustin, Ferdinand Heyn, Stella Ullrich, Ute Sandaradura de Silva, Marie-Christine Albert, Viktoria Linne, Maike Schlotz, Philipp Schommers, Elisabeth Pracht, Carola Horn, Isabelle Suarez, Alexander Simonis, Lea Katharina Picard, Alexander Zoufaly, Christoph Wenisch, Gerd Fätkenheuer, Henning Gruell, Florian Klein, Michael Hallek, Henning Walczak, Jan Rybniker, Sebastian J. Theobald, Clara Lehmann

**Affiliations:** ^1^Department I of Internal Medicine, Medical Faculty and University Hospital Cologne, University of Cologne, Cologne, Germany; ^2^Center for Molecular Medicine Cologne (CMMC), Faculty of Medicine and University Hospital Cologne, University of Cologne, Cologne, Germany; ^3^German Center for Infection Research (DZIF), Bonn-Cologne, Germany; ^4^Cologne Excellence Cluster on Cellular Stress Responses in Aging-Associated Diseases (CECAD), University of Cologne, Cologne, Germany; ^5^Institute for Biochemistry, Faculty of Medicine and University Hospital Cologne, University of Cologne, Cologne, Germany; ^6^Institute of Virology, Faculty of Medicine and University Hospital Cologne, University of Cologne, Cologne, Germany; ^7^Department IV of Internal Medicine, Klinik Favoriten, Vienna Healthcare Group, Vienna, Austria; ^8^Faculty of Medicine, Sigmund Freud University, Vienna, Austria; ^9^Centre for Cell Death, Cancer, and Inflammation (CCCI), UCL Cancer Institute, University College London, London, United Kingdom

**Keywords:** post-COVID syndrome, long COVID, immunosuppression, immune activation, immunological fingerprint, PCS

## Abstract

**Background:**

Symptoms lasting longer than 12  weeks after severe acute respiratory syndrome coronavirus type 2 (SARS-CoV-2) infection are called post-coronavirus disease (COVID) syndrome (PCS). The identification of new biomarkers that predict the occurrence or course of PCS in terms of a post-viral syndrome is vital. T-cell dysfunction, cytokine imbalance, and impaired autoimmunity have been reported in PCS. Nevertheless, there is still a lack of conclusive information on the underlying mechanisms due to, among other things, a lack of controlled study designs.

**Methods:**

Here, we conducted a prospective, controlled study to characterize the humoral and cellular immune response in unvaccinated patients with and without PCS following SARS-CoV-2 infection over 7 months and unexposed donors.

**Results:**

Patients with PCS showed as early as 6 weeks and 7 months after symptom onset significantly increased frequencies of SARS-CoV-2-specific CD4^+^ and CD8^+^ T-cells secreting IFNγ, TNF, and expressing CD40L, as well as plasmacytoid dendritic cells (pDC) with an activated phenotype. Remarkably, the immunosuppressive counterparts type 1 regulatory T-cells (TR1: CD49b/LAG-3^+^) and IL-4 were more abundant in PCS^+^.

**Conclusion:**

This work describes immunological alterations between inflammation and immunosuppression in COVID-19 convalescents with and without PCS, which may provide potential directions for future epidemiological investigations and targeted treatments.

## Introduction

While the majority of patients recover from coronavirus disease 2019 (COVID-19) caused by severe acute respiratory syndrome coronavirus 2 (SARS-CoV-2) without any apparent sequelae, a significant proportion of patients experience long-term sequelae, which is referred to as post-COVID syndrome (PCS) (1–5). According to the World Health Organization, 10–20% of people with COVID-19 report persistent or new symptoms representing a major issue for global health care (6, 7). Interestingly, occurrence of PCS is independent of disease severity and can be observed after both mild and severe COVID-19 (8, 9).

Until now, the pathophysiological mechanisms of PCS in terms of a post-viral syndrome are not well understood, complicating the identification of PCS patients and the approach of causal therapies. However, it is particularly important to develop a better understanding of the underlying mechanisms, which contribute to the development of PCS and may facilitate the identification of patients at risk. This includes, among other things, the need to investigate the cellular and humoral immune responses in the context of PCS (10). Notably, alterations in T-cell activation and function are known to play a role in post-acute viral syndromes (11, 12). In this context, changes in immune responses following SARS-CoV-2 infection including dysfunction of T-cells as well as B-cells (13, 14) have recently been reported. Furthermore, dysregulations in CD4^+^ and CD8^+^ T-cells such as increased autoantibody levels and altered distribution of memory cell subpopulations were associated with PCS (15, 16). However, the pattern of T-cell distribution, activation and exhaustion and its correlation to PCS are not yet fully understood.

To uncover the immunological imbalance connected to PCS, we carried out a prospective, controlled study to characterize the humoral and cellular immune response in unvaccinated patients with PCS following SARS-CoV-2 infection.

## Materials and methods

### Study design and sample preparation

**Figure 1:**
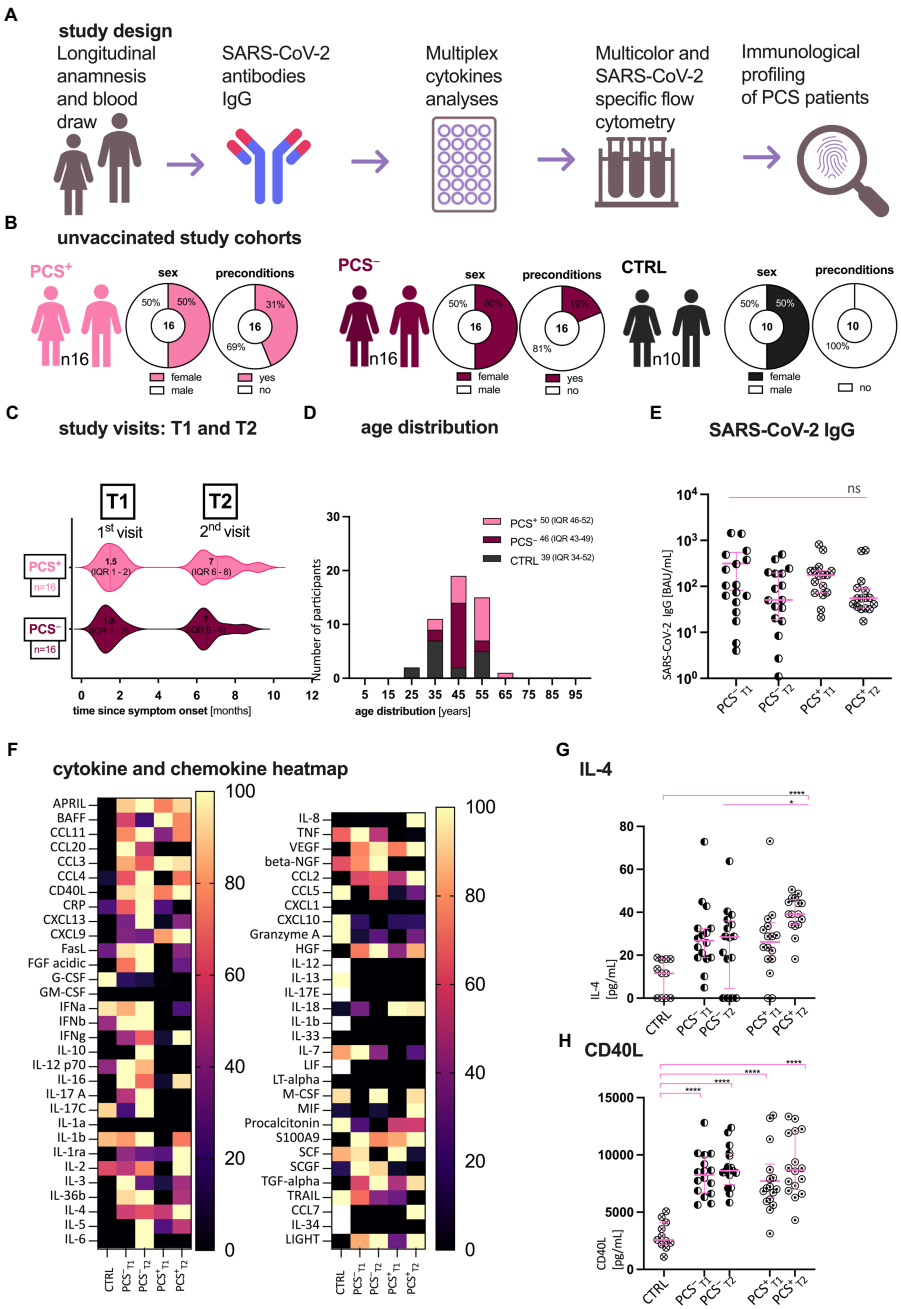
Patient characteristics, study design, and first serological assessment. **(A)** Study design; **(B)** Clinical characteristics of patients with (PCS^+^) and without (PCS^−^) post-COVID syndrome (PCS) and healthy volunteers (CTRL); **(C)** Study visits in months after symptom onset. **(D)** Age distribution of the cohorts. Data are expressed as absolute, percentage, or median (IQR), respectively. **(E)** Distribution of SARS-CoV-2 immunoglobulin G (IgG; BAU/mL); **(F)** Multiplex Luminex cytokine in CTRL, PCS^−^, and PCS^+^ at T1 and T2, respectively. Heatmap of normalized, median cytokine ratios [%]. **(G,H)** Exemplary plots of cytokine distribution (pg/mL): **(G)** IL-4, **(H)** CD40L. PCS, post-COVID syndrome; PCS^+^, patients with PCS; PCS^−^, patients without PCS; CTRL, control; *n*, number; pg., picogram; mL, milliliter; and IQR, interquartile range. Data information: For statistical analysis, normality was assessed by Shapiro–Wilk or Kolmogorov–Smirnov test, respectively. If non-parametrically distributed, Kruskal-Wallis tests with Dunn’s multiple comparisons and—if parametrically distributed—ANOVA with *post hoc* tests were used, as applicable. Detailed statistical information and all abbreviations used are shown in Supplementary Table 2. *p* < 0.05 shows statistical significance: ^*^p ≤ 0.05, ^**^p ≤ 0.01, ^***^p ≤ 0.001, ^****^p ≤ 0.0001, and ns, not significant.

Patients from the PCS outpatient clinic of the University Hospital Cologne (UHC) were included in our longitudinal study according to the WHO definition of PCS (17). Patients presented to our outpatient clinic shortly after the onset of each symptom, having been quarantined at home from April to June 2020 and continued to be monitored thereafter. Three unvaccinated cohorts were enrolled in our study during a matched timeframe (April 2020–December 2020): Unvaccinated SARS-CoV-2 convalescents with (PCS^+^, *n* = 16) and without (PCS^−^, *n* = 16) PCS at 6 weeks (T1) and 7 months (T2) after symptom onset (T0) and healthy corona-naïve controls (CTRL, *n* = 10). PCS was defined by one or a number of newly emerged symptoms, such as fatigue, dyspnea, anosmia, or ageusia at T2 that lasted for at least 2 months and could not be explained by an alternative diagnosis. These definition met and meets international guidelines (1, 17, 18). COVID-19 convalescents were included as follows: (1) age ≥ 18 and (2) confirmed SARS-CoV-2 infection by polymerase chain reaction testing in swab or sputum 3 months prior to study enrollment. The study was approved by the Institutional Review Board of the UHC (16_054 and 20_1187). According to the WHO progression scale (19), our study cohorts consisted of outpatients not needing oxygen support nor further assistance (WHO grade I-II, Supplementary Table 1). In addition, none of the participants received prior COVID therapy. At visits, patients completed standard questionnaires querying medical history and individual symptoms. Subsequently, clinical assessment of COVID-19 convalescents was performed and evaluated by a trained physician after additional anamnesis, physical examination and functional diagnostics. In addition, at all time points, blood samples were collected. Peripheral blood mononuclear cells (PBMC) were separated using density centrifugation on Ficoll gradient (Biochrom, L 6115). PBMC and additional serum and plasma samples were kept frozen at −180°C for future analysis. Subsequently, immunological profiling was performed on blood and serum samples in five steps (Figure 1A).

### Detection of SARS-CoV-2-specific immunoglobulin G

Immunoglobulin G (IgG) against the receptor binding domain (RBD) of the SARS-CoV-2 spike protein were measured by the SARS-CoV-2 IgG II Quant assay provided by Abbott on the Alinity i (Abbott, Abbott Park, IL, United States). IgG titers were reported in Binding Antibody Units per milliliter (BAU/mL) according to manufacturer’s instructions.

### Multiplex cytokine and chemokine analysis

Cytokine and chemokine analyses were performed by Luminex Discovery Assays (R&D Biotechne, Minneapolis, MN, United States) with the indicated analytes according to the manufacturer’s instructions. Cytokines were measured with Luminex 200 xMAP system. All abbreviations used are listed in Supplementary Table 2.

### Flow-cytometric analysis of T-cell subpopulations

The frequency and function of CD4^+^ T-cell subpopulations [CD3^+^CD4^+^ (T_CD4_), CD4^+^CD45RO^+^ memory T-cells (T_M_), CCR7 ± CD27 ± memory subsets (T_CM_: T central memory, T_EM_: T effector memory, and T_TM_: T transitional memory), and CD4^+^CD45RO^−^ antigen-naïve cells (T_N_)] was performed by flow cytometry using a BD FACSCanto. Detailed information on the antibodies and dilutions used in addition to the gating strategy is provided in Supplementary Figure S2. Furthermore, frequency and function of plasmacytoid dendritic cells (pDC BDCA2^+^CD123^+^) and type 1 regulatory T-cells (TR1 CD49b^+^LAG3^+^) were analyzed (Supplementary Figure S2), as were expression (geometric mean fluorescence intensity, gMFI) of T-cell activation and exhaustion markers programmed cell death protein 1 (PD-1), chemokine receptor 3 (CXCR3), and human leukocyte antigen DR (HLA-DR).

### SARS-CoV-2-reactive CD4^+^ and CD8^+^ T-cell interferon-γ, tumor necrosis factor, and CD40 ligand assay

Human PBMC were incubated for 6 h with the SARS-CoV-2-specific protein S1 (PS1) or left unstimulated (assay control) according to manufacturer’s instructions (Miltenyi, Biotec, Bergisch Gladbach, Germany). After pregating on CD3 as well as CD4 and CD8, respectively, frequencies of interferon-γ (IFNγ), tumor necrosis factor (TNF), and CD40L were assessed following stimulation. Samples were analyzed using BD Canto (Supplementary Figure S5).

### Statistical methods

The statistical analysis was performed using GraphPad Prism software v.9 (GraphPad, San Diego, CA, United States). Normality was assessed by Kolmogorov–Smirnov or Shapiro–Wilk test, respectively. *p* values of 0.05 and lower were considered as statistically significant. To test for significant differences in parametric and non-parametric distributions, either one-way ANOVA with *post hoc* analyses or Kruskal-Wallis tests with Dunn’s multiple comparisons were performed, as applicable. Here, *p* values were adjusted accordingly. Detailed statistical results are stated in Supplementary Table 2. Statistical parameters (value of *n*, statistical calculation, etc.) are stated in the figure legend. If not otherwise stated, all variables are represented as the median with the interquartile range (IQR).

## Results

### Declining SARS-CoV-2-specific antibody titers in unvaccinated patients

The demographic and clinical characteristics of enrolled patients are presented in Figures 1B–D and Supplementary Table 1. While gender and age distribution were comparable in all cohorts, PCS^+^ had more pre-existing preconditions, such as hypertension, diabetes or other metabolic diseases (PCS^+^ 44% > PCS^−^ 19% > CTRL). Notably, the incidence of concomitant symptoms decreased in PCS^+^ over time (T1 93.75%, T2 37.5%). Also, we determined SARS-CoV-2-specific immunoglobulin G (IgG) titers against the receptor binding domain (RBD) of the SARS-CoV-2 spike protein in plasma (Figure 1E). Here, a non-significant decline in both unvaccinated PCS^+^ and PCS^−^ was observed over time [*BAU/mL*; mean ± standard error of mean (SEM); PCS^−^ T1 315 ± 110, T2 125 ± 38; PCS^+^ T1 216 ± 54, T2 122 ± 47]. At both time points however, no significant difference in antibody titers were found (PCS^−^ ∼ PCS^+^), concluding that in this small cohort the level of RBD-specific SARS-CoV-2 antibody titers is not an indicator for the identification of PCS.

### Multiplex cytokine and chemokine analysis: Increase of IL-4 in PCS^+^

Considering that there might be a characteristic pattern of cytokines and chemokines in PCS^+^ we performed a multiplex luminex analysis of 61 cytokines/chemokines (Figure 1F; Supplementary Figure S1; Supplementary Table 2). Apart from IL-4 (*p* = 0.02, Supplementary Figure S1A), no significant increases were detected between PCS^+^ and PCS^−^ for all tested cytokines or chemokines, respectively. Hence, PCS^+^ could not be identified by a distinct cytokine pattern alone. However, when we compared the PCS^+^ with the controls, we were able to show an increase of CD40 ligand (CD40L, Figure 1H) and interleukin-4 (IL-4, Figure 1G), (CTRL vs. PCS^+:^ CD40L: T1 *p* < 0.0001, T2 *p* < 0.0001; IL-4: T1 *p* > 0.05, T2 *p* < 0.0001). In summary, IL-4 showed the most significant increase in PCS^+^ versus PCS^−^ and CTRL.

### CD4^+^ memory T-cell profiling: Increased frequency of transitional memory and decreased central memory CD4^+^ T-cells in PCS^+^

**Figure 2:**
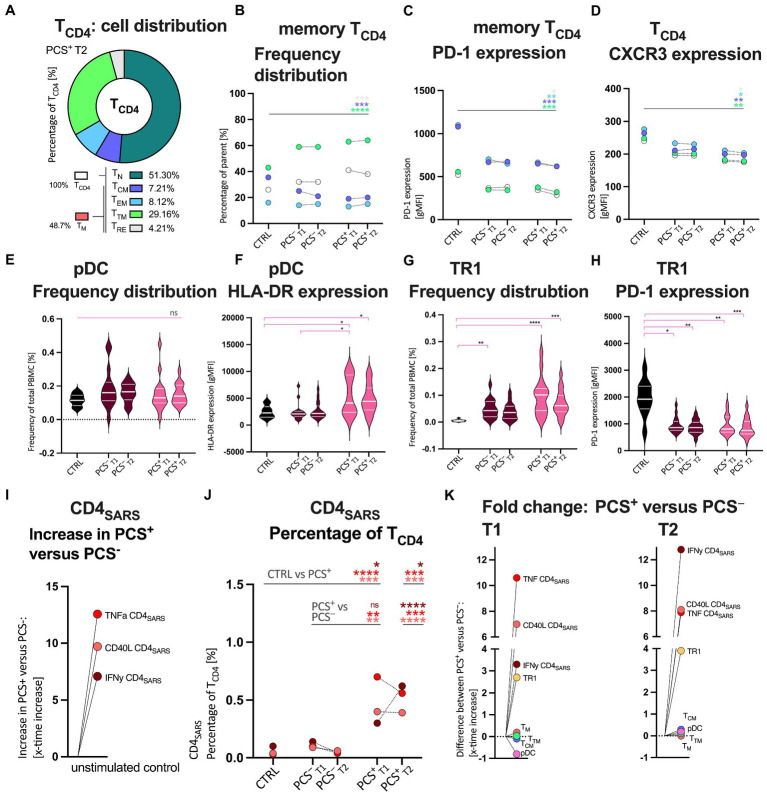
Quantitative and qualitative attributes of CD4^+^ T-cells (T_CD4_), Bystander cells, and protein-S1 (PS1)-stimulated SARS-CoV-2 specific CD4^+^ (CD4_SARS_) in CTRL, PCS^−^, and PCS^+^ at T1 and T2, respectively. **(A)** Exemplary distribution of all T_CD4_ in PCS^+^. Percentage of T_CD4_ (%). Color legends also apply to **B–D**. **(B)** Median frequency distribution of T_CD4_ and its memory subpopulations by cohort. Percentage of parent (%). **(C)** T_CD4_ and its memory subpopulations: Median expression of PD-1 (gMFI); **(D)** T_CD4_ and its memory subpopulations: Median expression of CXCR3 (gMFI). **(E)** Plasmacytoid dendritic cells (pDC): Frequency distribution (%). **(F)** pDC: Expression of human leukocyte antigen DR (HLA-DR; gMFI); **(G)** Type 1 regulatory T-cells (TR1): Frequency distribution (%) **H** TR1: Expression of PD-1 (gMFI). Distribution of distinct CD4_SARS_ in **I** unstimulated assay controls and **J** after PS1-stimulation in all cohorts (%). (**K**; i) Increased frequencies of PS1-stimulated CD4_SARS_, CD8_SARS_ (see Supplementary Figure S6), and TR1 in PCS^+^ versus PCS^−^ at T1 and T2, respectively. T_CD4_, CD4^+^ T-cells; T_N_, antigen-naive T-cells; T_M_, memory T-cells; T_CM_, central memory T-cells; T_EM_, effector memory T-cells; T_TM_, transitional memory T-cells; T_RE_, remainder memory T-cells; PCS, post-COVID syndrome; PCS^+^, patients with PCS; PCS^−^, patients without PCS; CTRL, control; CTRL_vac_, vaccinated controls; T1, first visit; T2, second visit; PBMC, peripheral blood mononuclear cells; and gMFI, geometric mean fluorescence intensity. Data information: For statistical analysis, normality was assessed by Shapiro–Wilk or Kolmogorov–Smirnov test, respectively. If non-parametrically distributed Kruskal-Wallis tests and if parametrically distributed ANOVA were used, as applicable. Detailed statistical analyses (*post hoc* test and Dunn’s multiple comparisons) and individual data of patients of **(B,C,D)** are shown in Supplementary Figures S3, S4 and Supplementary Table 2. *p* < 0.05 shows statistical significance: ^*^p ≤ 0.05, ^**^p ≤ 0.01, ^***^p ≤ 0.001, ^****^p ≤ 0.0001, and ns, not significant.

Hence, due to key implication of CD4^+^ T-cells in the response to SARS-CoV-2 and the results of the cytokine and chemokine analysis, we examined, CD4^+^ T-cells (T_CD4_, Figures 2A–D; Supplementary Figures S2–4) and the relevant memory T-cells subpopulations (T_CM_: T central memory, T_EM_: T effector memory, and T_TM_: T transitional memory) as well as the T-cell activation and exhaustion markers PD-1, CXCR3, and HLA-DR by flow cytometry. While Figure 2A displays the exemplary distribution of all T_CD4_ in PCS^+^, Figure 2B shows the detailed frequencies of T_CD4,_ but primarily its memory subpopulations. Here, frequencies of T_CD4_ were significantly increased in PCS^+^ when compared to CTRL and PCS^−^ at T1 (CTRL *p* = 0.0071; PCS^−^ *p* = 0.0180, Supplementary Figure S3A). In contrast to significantly higher frequencies of transitional memory T-cells (T_TM_; T1 *p* < 0.0001, T2 *p* < 0.0001, Figure 2B and Supplementary Figure S3F), central memory T-cells (T_CM_) were significantly reduced in PCS^+^ when compared to CTRL (T1 *p* = 0.0023, T2 *p* < 0.0001, Figure 2B and Supplementary Figure S3E). Notably, no significant differences were detected between PCS^+^ and PCS^−^ (Supplementary Figures S3E,F). Finally, expression of both programmed cell death protein 1 (PD-1, Figure 2C and Supplementary Figures S4A–C) and CXCR3 (Figure 2D; Supplementary Figures S4D–F) were highest in CTRL (CTRL > PCS^+^ = PCS^−^), indicating that the CD4^+^ subpopulations were neither exhausted nor activated.

### Increased pDC activation and high frequencies of TR1 in PCS^+^

In order to better understand the immunomodulatory capacities of interactions between innate and adaptive immunity (20, 21), we assessed proinflammatory cells such as plasmacytoid dendritic cells (pDC) and immunosuppressive type 1 regulatory T-cells (TR1) by flow cytometry (Figures 2E–H; Supplementary Figures S2C,E). While pDC were almost equally distributed between groups (Figure 2E), TR1 were 2.7 and 3.9-fold higher in PCS^+^, respectively [Figure 2G; PCS^+^ versus CTRL: T1 *p* < 0.0001, T2 *p* = 0.0003; fold change (fc) PCS^+^ versus PCS^−^: T1 2.7 T2 3.9, n.s.]. However, the observed increase lacked statistical significance, likely due to limited number of cases. PD-1 expression on TR1 cells was significantly reduced in both PCS^+^ (T1 *p* = 0.005, T2 *p* = 0.0007) and PCS^−^ (T1 *p* = 0.02, T2 *p* = 0.0038) versus CTRL (Figure 2G; Supplementary Figure S4H). Again, no relevant difference was detected between PCS^+^ and PCS^−^ (fc; T1 0.03, T2 0.06). However, HLA-DR expression on pDC was increased in PCS^+^ versus PCS^−^ (Figure 2F and Supplementary Figure S4G; T1 *p* = 0.05, T2 *p* = 0.06) and versus CTRL (Figure 2F; Supplementary Figure S4G; T1 *p* = 0.03, T2 *p* = 0.04).

### SARS-CoV-2-specific T-cells producing IFNγ, TNF, and expressing CD40L were more often observed in PCS^+^

As we observed an altered CD4^+^ memory T-cell profiling we aimed to examine functionality of SARS-CoV-2-specific T-cells. To this end, we evaluated SARS-CoV-2-specific T-cell responses by intracellular cytokine staining of interferon γ (IFNγ), tumor necrosis factor alpha (TNF), and CD40 ligand (CD40L) following SARS-CoV-2-specific protein S1 (PS1) stimulation (CD4_SARS_; Figures 2I,J and CD8_SARS_; Supplementary Figure S6). We found 5–12-times higher frequencies of CD4_SARS_ secreting IFNγ, TNF, and expressing CD40L in unstimulated PBMCs (assay controls) of PCS^+^ compared to PCS^−^, (Figure 2I). After PS1-stimulation, frequencies of CD4_SARS_ and CD8_SARS_, respectively, were significantly highest in PCS^+^ when compared to PCS^−^ and controls (PCS^−^: CD4_SARS_, Figure 2J, T2 IFNγ *p* < 0.0001, TNF *p* = 0.0006, CD40L *p* < 0.0001; CD8_SARS_, Supplementary Figure S6, T2 IFNγ *p* = 0.048, TNF *p* = 0.0008, CD40L *p* = 0.0009; CTRL: CD4_SARS_, Figure 2J, T1 IFNγ *p* = 0.04, TNF *p* < 0.0001, CD40L *p* = 0.0023; T2 IFNγ *p* = 0.01, TNF *p* = 0.0008, CD40L *p* = 0.0053; CD8_SARS_, Supplementary Figure S6, CTRL: T1 IFNγ *p* = 0.004, TNF *p* = 0.0005, CD40L *p* = 0.0331; T2 IFNγ *p* > 0.05, TNF *p* < 0.0001, CD40L *p* = 0.0002). Notably, SARS-CoV-2-specific CD4 and CD8 T-cells secreting IFNγ, TNF and expressing CD40L were 2–8-times more frequent in PCS^+^ than in PCS^−^ at T1 and T2 (fold change, *x*-times higher; IFNγ T1 3.3, T2 12.8; TNF T1 10.6, T2 7.9; CD40L T1 7.0, T2 8.1).

**Figure 3:**
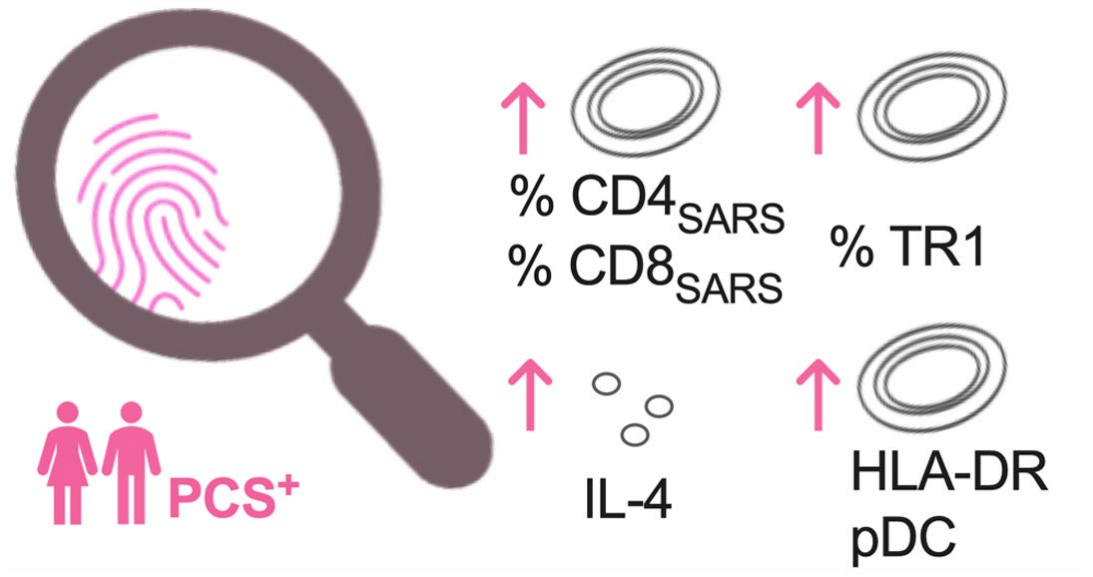
Most pronounced immune alterations in PCS patients at week 6 and month 7 after symptom onset. Increased frequencies of protein-S1-stimulated SARS-CoV-2 specific CD4^+^ (CD4_SARS_) and CD8^+^ T-cells (CD8_SARS_), type 1 regulatory T cells (TR1), levels of interleukin 4 (IL-4), and HLA-DR expression on plasmacytoid dendritic cells (pDC) in PCS patients when compared to convalescents without PCS and controls. PCS, post-COVID syndrome; PCS^+^, PCS patients; and HLA-DR, human leukocyte antigen DR.

In summary, in PCS^+^, we observed an increased frequency of SARS-CoV-2-specific CD4^+^ T-cell and CD8^+^ T-cell responses secreting IFNγ, TNF, and CD40L both in unstimulated PBMC and after PS1 stimulation (Figure 2K). Also, frequencies of TR1 and activation status of pDC and levels of IL-4 were highest in PCS^+^ (Figures 2K, 3).

## Discussion

Reliable diagnosis of PCS would help to direct patient care to where it is most needed. Here, we set out to determine whether comparative humoral and cellular immune profiling of unvaccinated COVID-19 convalescents with and without PCS and control individuals without any SARS-CoV-2 infection and SARS-CoV-2 vaccination could allow the identification of a specific immune profile that could distinguish patients with PCS from those without PCS in a consistent way.

In line with previous studies (13, 22), our analysis revealed that neither levels of SARS-CoV-2 IgG or cytokines nor quantitative and qualitative features of CD4^+^ T-cells alone allowed an identification of PCS^+^. Also, after completing a SARS-CoV-2-specific cellular immune and bystander immune assessment, a clear distinction between COVID-19 convalescents with and without PCS was not possible. However, distinct patterns of altered immune responses were most apparent in PCS^+^ as early as 6 weeks after symptom onset, which persisted until the seventh month after. Primarily, this pattern consisted of increased frequencies of SARS-CoV-2-specific CD4^+^T-cells secreting IFNγ, TNF, and expressing CD40L and CD8_SARS_ TR1, activated pDC and elevated levels of IL-4 (Figures 2J, 3). These findings indicate immunological alterations of both immune activation (CD4_SARS_ and CD8_SARS_ and activated pDC) versus immunosuppression (TR1 and IL-4) in both innate and adaptive immunity.

Elevated immune activation is common in many post-viral syndromes following infection with Epstein–Barr-virus (EBV), varicella-zoster virus (VZV), herpes simplex virus (HSV), and cytomegalovirus (CMV) and is displayed by pathogen-specific T-cells (23, 24). In addition, previous or recent studies showed SARS-CoV-2-specific memory T-cell responses in COVID-19 convalescents up to 3–8 months after infection (25, 26) and vaccination (27–29), respectively. However, the frequency distribution and the roles of CD4_SARS_ and CD8_SARS_ T- cells in PCS remained poorly understood. We show higher frequencies of CD4_SARS_ and CD8_SARS_ in PCS^+^ at 6 weeks and 7 months after symptom onset, in PCS^+^ compared to PCS^−^ (Figures 2K 3). The increased frequency of SARS-CoV-2-specific CD4^+^ T-cells secreting IFNγ, TNF, and expressing CD40L may lead in total to an increase in these cytokines and subsequently might contribute to an elevated systemic inflammation. In our study, elevated immune activation is further supported by a stronger activated phenotype (HLA-DR) of pDC in PCS^+^ (Figure 2F), which is in line with data of Australian colleagues (13). pDC are important in controlling viral replication through the induction of innate immune response but are also involved in inducing immune activation (30).

Furthermore, in unstimulated test controls of PCS^+^, a 5–12-fold increase in the frequency of CD4_SARS_/CD8_SARS_ secreting IFNγ and TNF and expressing CD40L is observed (Figure 2I). This increased cytokine secretion without additional stimulation suggests increased baseline inflammation in PCS^+^, which could be understood as a distinct, immunological awakening in response to uncontrolled SARS-CoV-2-dependent immune alterations most pronounced in PCS^+^. Indeed, inflammation is highly implicated in the mechanism of the post-COVID-19 condition and its neurological sequelae. Suitably, CD40L, which is transiently expressed on T-cells and other non-immune cells under inflammatory conditions (31), was augmented in PCS^+^ (Figure 1G). A consequence of the chronic immune activation may be the described altered distributions of memory T-cells (T_M_) and subpopulations (T_CM_, T_TM_) which play a crucial role in viral clearance during reinfection and evolution of immunity (Figures 2A,B). Thereby, the T_TM_, constituted the largest proportion in PCS^+^ (Figure 2B). However, in line with previous data on severe COVID-19 cases during the acute phase of infection (32, 33), frequencies of T_CM_ were lowest in PCS^+^ when compared to PCS^−^ and CTRL (Figure 2B). Finally, it is noteworthy that during other chronic infections caused by CMV, EBV or human immunodeficiency virus (HIV), similar changes in the distributions of memory T-cells can be observed (34, 35).

In response to the increased inflammation observed in PCS, the immune system appears to initiate immunosuppressive countermeasures. In particular, IL-4 (Figure 1I) and immunosuppressive TR1 (Figure 2G) were upregulated in PCS^+^, possibly indicating an endogenous loop actively counteracting enhanced inflammation and further chronic immune activation. Elevated levels of IL-4 in PCS patients are in agreement with data found by Klein and colleagues (32). Furthermore, TR1 are antigen-specific adaptive bystander cells, able to reestablish tolerance in immune-mediated diseases (36). It remains unclear whether the observed increase has beneficial or detrimental effects on the course. On the one hand, high frequency of TR1 could be harmful, as they might reduce SARS-CoV2-specific responses and thus contribute to viral persistence. On the other hand, increasing TR1 levels observed in PCS could be beneficial by suppressing generalized chronic immune activation, especially through the inhibition of activated CD4.

Finally, it should be noted that for all other variables if any significant differences were observed compared to healthy controls, this was independent of PCS status (COVID-19 convalescents: PCS^+^ and PCS−). Hence, according to this study, most parameters assessed were not suitable for PCS identification. There might be several reasons for this. Firstly, since no specific diagnostic biomarkers have been identified so far, the diagnosis of PCS must be made by clinical investigation according to the WHO definition (17). In this context, the complexity is to distinguish a PCS in the sense of direct biological consequences of the infection with SARS-CoV-2 (post-viral syndrome) from post-traumatic stress disorders.

Our study has further limitations. It is therefore possible that patients have been misclassified in the group of post-viral syndromes which may have led to a lack of clear significant results. Therefore, a deeper distinction between symptoms that are reliably directly SARS-CoV-2-related and are not caused by the exceptional pandemic burden (37–40) is imperative. Second, group size was probably too small to draw statistically significant conclusions. Third, given the timing of infection in early 2020 we assume that all patients were infected with the Wuhan wild-type in the period before vaccination which makes pre-existing immunity in patients highly unlikely. However, it has to be noted that post-viral sequelae after infection with current Omicron variants might be different. Fourth, PCS^+^ were slightly older and had more pre-existing conditions when compared to PCS^−^ in our study which in turn could have contributed to enhanced basal inflammation of PCS+. Fifth, evaluation of T-cell exhaustion and activation markers were not assessed on SARS-CoV-2 reactive T-cells. Also, SARS-CoV-2 IgG titers were not available in matched control groups and during acute phase of disease (T0). Still, a major advantage of this study is that it is a prospective and longitudinal observation of unvaccinated and thus virologically virgin individuals with a matched control group, which, to our knowledge, has rarely been carried out before. However, the vast majority of the current population in 2023 is equipped with pre-existing immunity due to prior infections or vaccinations. In those, SARS-CoV-2-related immune alterations might develop differently as described here.

After all, in the current study, we conducted a prospective, controlled study to characterize immune response in unvaccinated patients with and without PCS following SARS-CoV-2 infection over 7 months. We describe immunological alterations between inflammation and immunosuppression in COVID-19 convalescents with and without PCS. The conclusion needs to be validated by larger studies based on a systematic blood collection protocol and other SARS-CoV-2 variants. Despite this limitation, our current analysis provides valuable information regarding immunological profiling of PCS. These data contribute to our basic understanding and identification of PCS as a post-viral syndrome, facilitating the development of effective targeted therapies and epidemiological investigations.

## Data availability statement

The original contributions presented in the study are included in the article/Supplementary material, further inquiries can be directed to the corresponding author.

## Ethics statement

The study was conducted in accordance with the Declaration of Helsinki, and approved by the Institutional Review Board of University Hospital Cologne (16-540 and 20-1187_1). The patients/participants provided their written informed consent to participate in this study.

## Author contributions

MA, FH, SU, and CL: conceptualization, methodology, investigation, data curation, and writing—original draft preparation. MA, CH, and CL: software and validation. MA, FH, SU, ST, M-CA, and US: formal analysis. MA, FK, HG, ST, AS, JR, M-CA, HW, AZ, CL, and US: resources. MA, FH, SU, US, M-CA, VL, MS, PS, EP, CH, IS, AS, LP, AZ, CW, GF, HG, FK, MH, HW, JR, ST, and CL: writing—review and editing. MA, FH, and SU: visualization. CL, JR, FK, GF, and MH: supervision. CL and JR: project administration. MA, CL, GF, JR, HW and FK: funding acquisition. All authors contributed to the article and approved the submitted version.

## Funding

This research was funded by COVIM as part of Netzwerk Universitätsmedizin (NUM, “NaFoUniMedCovid19,” FKZ: 01KX2021), the Federal Ministry of Education and Research Germany (BMBF, FKZ 01EP2106), and the German Center for Infection Research (DZIF TTU HIV 04.820). For the remaining authors, none are declared. PS received funding from German Research Foundation (DFG, Project No: 495793173). HW is supported by Alexander von Humboldt Foundation, a Wellcome Trust Investigator Award (214342/Z/18/Z) and a collaborative research center grant (SFB1403–414786233) funded by the Deutsche Forschungsgemeinschaft (DFG, German Research Foundation). AS received funding from the German Federal Ministry of Education and Research (BMBF) (01KI2108).

## Conflict of interest

The authors declare that the research was conducted in the absence of any commercial or financial relationships that could be construed as a potential conflict of interest.

## Publisher’s note

All claims expressed in this article are solely those of the authors and do not necessarily represent those of their affiliated organizations, or those of the publisher, the editors and the reviewers. Any product that may be evaluated in this article, or claim that may be made by its manufacturer, is not guaranteed or endorsed by the publisher.
