# Supplementary material for: Immunological fingerprint in coronavirus disease-19 convalescents with and without post-COVID syndrome
Source: Front Med (Lausanne). 2023 Apr 24;10:1129288. doi: 10.3389/fmed.2023.1129288 (PMC10165999; doi:10.3389/fmed.2023.1129288)
Supplement: Supplementary file 1 [file Data_Sheet_1.PDF]

		PCS+ (n=16)	PCS− (n=16)	CTRL (n=10)
	**median age (IQR),** years	50 (47-52)	46 (44-49)	43 (25-57)
	<50 years, n (%)	10 (62.5%)	14 (87.5%)	6 (60%)
	>50 years, n (%)	6 (37.5%)	5 (31.3%)	4 (40%)
	**sex,** n (%)			
	male	8 (50%)	8 (50%)	5 (50%)
	female	8 (50%)	8 (50%)	5 (50%)
	**WHO progression scale,** n (%)			
	**I - II** (ambulatory mild disease, independent)	16 (100%)	16 (100%)	n.a.
	**III** (ambulatory mild disease, assistence needed)	0	0	n.a.
	**IV-X** (hospitalised: moderate or severe disease)	0	0	n.a.
	oxygen support	0	0	n.a.
	COVID-19 vaccination	0	0	0
	**pre-existing conditions,** n (%)	5 (31.25%)	3 (18.75 %)	0 (0 %)
	hypertension	2 (12.5%)	1 (6.25%)	0 (0 %)
	diabetes	1 (6.25%)	0 (0 %)	0 (0 %)
	chronic lung disease	1 (6.25%)	0 (0 %)	0 (0 %)
	autoimmune disease	2 (12.5%)	0 (0 %)	0 (0 %)
	malignancies	0 (0%)	0 (0 %)	0 (0 %)
	congenital heart defects	0 (0 %)	2 (12.5%)	0
symptoms during acute phase (T0)	**PCS defining symptoms,** n (%)	15 (93.75%)	10 (62.5%)	n.a.
	anosmia	15 (93.75%)	9 (56.25%)	n.a.
	ageusia	13 (81.25%)	9 (56.25%)	n.a.
	fatigue	0 (0 %)	0 (0 %)	n.a.
	dyspnea	0 (0 %)	0 (0 %)	n.a.
	**concominant symptoms,** n (%)	15 (93.75%)	16 (100%)	n.a.
	median (IQR)	3 (2-4)	3 (2-4)	n.a.
	cough	12 (75%)	12 (75%)	n.a.
	rhinitis	8 (50%)	7 (43,75%)	n.a.
	headache	6 (37,5%)	8 (50%)	n.a.
	muscle pain	10 (62,5%)	10 (62,5%)	n.a.
	concentration disorder	0 (0%)	0 (0%)	n.a.
	fever	1 (6,25%)	8 (50%)	n.a.
	no symptoms	0	0	n.a.
symptoms at 2nd visit (median 7 months after initial symptoms)	**PCS defining symptoms T2,** n (%)	16 (100%)	0	n.a.
	anosmia	13 (81.25%)	0	n.a.
	ageusia	11 (68.25%)	0	n.a.
	fatigue	6 (37.5%)	0	n.a.
	dyspnea	5 (31.25%)	0	n.a.
	**concominant symptoms T2,** n (%)	6 (37.5%)	0	n.a.
	median (IQR)	0 (0-4)	0 (0)	n.a.
	cough	2 (12.5%)	0	n.a.
	rhinitis	0	0	n.a.
	headache	2 (12.5%)	0	n.a.
	muscle pain	2 (12.5%)	0	n.a.
	concentration disorder	4 (25%)	0	n.a.
	fever	0	0	n.a.
	no symptoms	0	16 (100%)	n.a.

Supplementary table 1. Patient characteristics. COVID-19, coronavirus disease 19; WHO, world health organisation; PCS, post-COVID syndrome; PCS+, patients with PCS; PCS−, patients without PCS; CTRL, unvaccinated controls; $CTRL_{vac}$, vaccinated controls; T0, symptom onset; T1, first visit; T2, second visit; IQR, interqaurtile range; n.a., not

variable	parametric distribution (SWT/KST))?	ANOVA/ KWT	PCS^+ T1 vs. CTRL	PCS^+ T2 vs. CTRL	PCS^- T1 vs. CTRL	PCS^- T2 vs. CTRL	PCS^+ T1 vs. PCS^- T1	PCS^+ T2 vs. PCS^- T2
percentage distribution (%)								
T_{CD4}	yes	**.0002**	**.0071**	.0620	.7920	.9996	**.0180**	**.0263**
T_M	yes	.9412	.9996	.9996	.9996	.9996	.9996	.9998
T_N	yes	**.0160**	**.0207**	.1670	.2338	.7145	.6632	.5378
T_{CM}	no	**.0002**	**.0023**	**< .0001**	.0796	**.0002**	> .9999	.9999
T_{EM}	no	.6143	> .9999	> .9999	> .9999	> .9999	> .9999	> .9999
T_{TM}	no	**< .0001**	**< .0001**	**< .0001**	**.0247**	**.0290**	.9426	.3423
TR1	yes	**< .0001**	**< .0001**	**.0003**	**.0072**	.0726	.7175	.8203
pDC	no	.3190	> .9999	> .9999	.6757	.6812	> .9999	> .9999
$CD4_{SARS}$ IFNγ	no	**< .0001**	**.0486**	**.0185**	.9999	.9999	.3774	**< .0001**
$CD4_{SARS}$ TNF	no	**< .0001**	**< .0001**	**.0008**	> .9999	> .9999	**.0058**	**.0006**
$CD4_{SARS}$ CD40L	no	**< .0001**	**.0002**	**.0005**	> .9999	> .9999	**.0085**	**< .0001**
$CD8_{SARS}$ IFNγ	no	**.0001**	**.0040**	.1845	.8362	> .9999	.5381	**.0408**
$CD8_{SARS}$ TNF	no	**< .0001**	**.0005**	**< .0001**	> .9999	> .9999	.0630	**.0008**
$CD8_{SARS}$ CD40L	no	**< .0001**	**.0331**	**.0002**	.1686	> .9999	> .9999	**.0009**
expression of (gMFI)								
PD-1 on T_{CM}	no	**.0002**	**.0010**	**< .0001**	**.0042**	**.0089**	.9999	.99999
PD-1 on T_{EM}	yes	**.0025**	**.0211**	**.0010**	**.0433**	**.0183**	> .9999	> .9999
PD-1 on T_{TM}	yes	**.0007**	**.0061**	**.0004**	**.0114**	**.0036**	> .9999	> .9999
CXCR3 on T_{CM}	no	**.0065**	**.0104**	**.0074**	**.0364**	**.0359**	> .9999	> .9999
CXCR3 on T_{EM}	no	**.0114**	**.0256**	**.0084**	.3810	.2506	> .9999	> .9999
CXCR3 on T_{TM}	no	**.0023**	**.0047**	**.0032**	**.0127**	**.0106**	> .9999	> .9999
PD-1 on TR1	no	**.0010**	**.0050**	**.0007**	**.0206**	**.0038**	> .9999	> .9999
HLA-DR on pDC	no	**.0008**	**.0379**	**.0415**	> .9999	> .9999	**.05**	.0651
levels (pg/ml)								
APRIL	yes	**.0015**	**.0222**	**.0032**	**.0032**	**.0023**	.9998	> .9999
BAFF	yes	**.0163**	**.0211**	.1935	.2975	.9810	.9365	.7305
CCL11	n.a.	n.a.	n.a.	n.a.	n.a.	n.a.	n.a.	n.a.
CCL20	n.a.	n.a.	n.a.	n.a.	n.a.	n.a.	n.a.	n.a.
CCL3	no	**.0172**	**.0265**	**.0187**	.3638	.6125	> .9999	> .9999
CCL4	no	**.0022**	> .9999	**.0160**	.4600	.1239	.6328	> .9999
CD40L	yes	**< .0001**	**< .0001**	**< .0001**	**< .0001**	**< .0001**	.9986	> .9999
CRP	yes	**.0005**	> .9999	> .9999	.1148	.0712	**.0210**	**.0231**
CXCL13	yes	.6599	> .9999	.9990	.8657	.9714	.9616	> .9999
CXCL9	no	.0748	> .9999	.1937	> .9999	> .9999	> .9999	.2419
FasL	yes	.6227	.9872	.9872	.9700	.9766	.8516	.9872
FGF acidic	no	.0891	> .9999	> .9999	> .9999	.7314	.5808	> .9999
G-CSF	no	.3565	> .9999	.4169	> .9999	> .9999	> .9999	> .9999
GM-CSF	n.a.	n.a.	n.a.	n.a.	n.a.	n.a.	n.a.	n.a.
IFNa	no	**.0003**	**.0003**	**.0009**	**.0027**	**.0061**	.9980	.9992
IFNb	no	.1050	> .9999	> .9999	> .9999	> .9999	.4225	.6974
IFNg	no	.0875	> .9999	.2427	> .9999	.7269	> .9999	> .9999
IL-10	no	**.0396**	> .9999	> .9999	> .9999	.3707	.8635	.1840
Il-12 p70	no	**.0006**	**.0070**	.0564	> .9999	> .9999	**.0070**	.0564
IL-16	no	**.0050**	> .9999	**.0199**	.0767	.0979	.4274	> .9999
IL-17A	no	.4441	> .9999	> .9999	> .9999	> .9999	> .9999	> .9999
IL-17C	no	**.0031**	**.0268**	0.0570	> .9999	> .9999	.3247	.1376
IL-1a	n.a.	n.a.	n.a.	n.a.	n.a.	n.a.	n.a.	n.a.
IL-1b	no	**.0413**	> .9999	> .9999	> .9999	> .9999	.1808	> .9999
IL-1ra	n.a.	n.a.	n.a.	n.a.	n.a.	n.a.	n.a.	n.a.
IL-2	no	**.0052**	.9603	> .9999	> .9999	> .9999	.5740	> .9999
IL-3	no	.0655	> .9999	.1142	.1004	.3804	> .9999	> .9999
IL-36b	no	.1038	> .9999	> .9999	.8996	.3183	> .9999	> .9999
IL-4	no	**< .0001**	.1575	**< .0001**	.0589	.1688	> .9999	**.0297**
IL-5	no	.7876	> .9999	> .9999	> .9999	> .9999	> .9999	> .9999
IL-6	no	.1825	> .9999	.8245	.9919	.3217	> .9999	> .9999
IL-8	no	**< .0001**	.2871	**< .0001**	.0635	**.0002**	> .9999	> .9999
TNF	no	.0623	.6437	**> .9999**	> .9999	> .9999	.1804	> .9999
VEGF	no	**.0441**	.7472	**.0289**	.5589	.1231	> .9999	> .9999
beta-NGF	no	.1332	> .9999	> .9999	> .9999	> .9999	> .9999	.3567
CCL2	no	**.0004**	.0987	**.0001**	.0665	**.0188**	> .9999	> .9999
CCL5	no	.7390	> .9999	> .9999	> .9999	> .9999	> .9999	> .9999
CXCL1	n.a.	n.a.	n.a.	n.a.	n.a.	n.a.	n.a.	n.a.
CXCL10	no	.9739	> .9999	> .9999	> .9999	> .9999	> .9999	> .9999
Granzyme A	no	**.0053**	**.0018**	.2741	**.0427**	.1818	> .9999	> .9999
HGF	no	**.0003**	.5872	**.0023**	**.0228**	**.0006**	> .9999	> .9999
Il-12	n.a.	n.a.	n.a.	n.a.	n.a.	n.a.	n.a.	n.a.
IL-13	n.a.	n.a.	n.a.	n.a.	n.a.	n.a.	n.a.	n.a.
IL-17E	n.a.	n.a.	n.a.	n.a.	n.a.	n.a.	n.a.	n.a.
IL-18	yes	**.0292**	.0554	.2078	.7139	.8113	.3084	.4913
IL-1b	n.a.	n.a.	n.a.	n.a.	n.a.	n.a.	n.a.	n.a.
IL-33	n.a.	n.a.	n.a.	n.a.	n.a.	n.a.	n.a.	n.a.
IL-7	yes	**.0185**	.1141	**.0107**	.6467	.3180	.9738	.7897
LIF	n.a.	n.a.	n.a.	n.a.	n.a.	n.a.	n.a.	n.a.
LT-alpha	n.a.	n.a.	n.a.	n.a.	n.a.	n.a.	n.a.	n.a.
M-CSF	no	**.0130**	.0936	> .9999	> .9999	> .9999	> .9999	> .9999
MIF	no	**.0002**	.3099	> .9999	.6165	> .9999	> .9999	> .9999
Procalcitonin	no	.7391	> .9999	> .9999	> .9999	> .9999	> .9999	> .9999
S100A9	no	**.0003**	**.0100**	**.0005**	**.0002**	**.0009**	> .9999	> .9999
SCF	no	.1160	> .9999	> .9999	> .9999	> .9999	> .9999	> .9999
SCGF	no	**.0168**	> .9999	> .9999	.3616	.3105	.5188	.1175
TGF-alpha	no	**.0020**	.8814	**.0051**	.5284	**.0054**	> .9999	> .9999
TRAIL	yes	**.0017**	**.0207**	**.0010**	.5168	.1358	.7402	.5285
CCL7	n.a.	n.a.	n.a.	n.a.	n.a.	n.a.	n.a.	n.a.
IL-34	n.a.	n.a.	n.a.	n.a.	n.a.	n.a.	n.a.	n.a.
LIGHT	no	**< .0001**	.2359	**< .0001**	**.0003**	**< .0001**	.3192	> .9999

Supplementary table 2. Detailed statistical analysis. Normality was assessed by Shapiro-Wilk (SWT) or Kolmogorov-Smirnov test (KST), respectively. If parametrically distributed, analysis of variance (ANOVA was performed). If non-parametrically distributed, Kruskal-Wallis test (KWT) was performed. p <0.05 shows statistical significance: * p ≤ 0.05, ** p ≤ 0.01, *** p ≤ 0.001, **** p ≤ 0.0001, and n.a., not applicable.

SARS-CoV-2, severe acute respiratory syndrome coronavirus type 2; PCS, post-COVID syndrome; PCS+, patients with PCS; PCS−, patients without PCS; CTRL, unvaccinated controls; T1, first visit; T2, second visit; gMFI, geometric mean fluorescence intensity; n.a., not applicable; T_{CD4}, CD4+ T-cells; T_N, antigen-naive T-cells; T_M, memory T-cells; T_{CM}, central memory T-cells; T_{EM}, effector memory T-cells; T_{TM}, transitional memory T-cells; pDC, plasmacytoid dendritic cells; TR1, type 1 regulatory T-cells; $CD4_{SARS}$, CD4 positive SARS-CoV-2-reactive T-cells; $CD8_{SARS}$, CD8 positive SARS-CoV-2-reactive T-cells; IFNγ, interferon-gamma; TNF, tumor necrosis factor alpha; CD40L, CD40 ligand; PD-1 programmed cell death protein 1; CXCR3, chemokine receptor 3; HLA-DR, human leukocyte antigen DR; APRIL, a proliferation-inducing ligand; BAFF, B-cell activating factor; CCL, chemokine ligand; CRP, C reactive protein; CXCL; chemokine ligand; FasL, Fas ligand; FGF acidic, fibroblast growth factor acidic; G-CSF, granulocyte colony-stimulating factor; GM-CSF, granulocyte/macrophage colony-stimulating factor; M-CSF, macrophage colony-stimulating factor; IFN, interferon; IL, interleukin, TNF, tumor necrosis factor; VEGF, vascular endothelial growth factor; beta-NGF, beta nerve growth factor; HGF, hepatocyte growth factor; LIF, leukemia inhibitory factor; LT-alpha, lymphotoxin-alpha, MIF, macrophage migration inhibitory factor; S100A9, S100 calcium-binding protein; SCF, stem cell factor; SCGF, stem cell growth factor; TGF-alpha, transforming growth factor-alpha; TRAIL, TNF-related apoptosis-inducing ligand; LIGHT, lymphotoxin-like inducible protein that competes with glycoprotein D for herpesvirus entry on T-cells.

A Detailed statistical analysis of 61 cytokines or chemokines, respectively

cytokine or chemokine	para-metric ?	1st ANOVA/ KWT	2nd post hoc: significant increase PCS⁺ vs. PCS⁻
APRIL	yes	***.0015***	no
BAFF	yes	***.0163***	no
CCL11	n.a.	*n.a.*	no
CCL20	n.a.	*n.a.*	no
CCL3	no	***.0172***	no
CCL4	no	***.0022***	no
CD40L	yes	*<.0001*	no
CRP	yes	***.0005***	no
CXCL13	yes	*.6599*	no
CXCL9	no	*.0748*	no
FasL	yes	*.6227*	no
FGF acidic	no	*.0891*	no
G-CSF	no	*.3565*	no
GM-CSF	n.a.	*n.a.*	n.a.
IFNa	no	***.0003***	no
IFNb	no	*.1050*	no
IFNg	no	*.0875*	no
IL-10	no	***.0396***	no
Il-12 p70	no	***.0006***	no
IL-16	no	***.0050***	no
IL-17A	no	*.4441*	no
IL-17C	no	***.0031***	no
IL-1a	n.a.	*n.a.*	n.a.
IL-1b	no	***.0413***	no
IL-1ra	n.a.	*n.a.*	n.a.
IL-2	no	***.0052***	no
IL-3	no	*.0655*	no
IL-36b	no	*.1038*	no
IL-4	no	*<.0001*	**yes**
IL-5	no	*.7876*	no
IL-6	no	*.1825*	no

cytokine or chemokine	para-metric ?	1st ANOVA/ KWT	2nd post hoc: significant increase PCS⁺ vs. PCS⁻
IL-8	no	<.0001	no
TNF	no	.0623	no
VEGF	no	***.0441***	no
beta-NGF	no	.1332	no
CCL2	no	***.0004***	no
CCL5	no	.7390	no
CXCL1	n.a.	n.a.	n.a.
CXCL10	no	.9739	no
Granzyme A	no	***.0053***	no
HGF	no	***.0003***	no
Il-12	n.a.	n.a.	n.a.
IL-13	n.a.	n.a.	n.a.
IL-17E	n.a.	n.a.	n.a.
IL-18	yes	***.0292***	no
IL-1b	n.a.	n.a.	n.a.
IL-33	n.a.	n.a.	n.a.
IL-7	yes	***.0185***	no
LIF	n.a.	n.a.	n.a.
LT-alpha	n.a.	n.a.	n.a.
M-CSF	no	***.0130***	no
MIF	no	***.0002***	no
Procalcitonin	no	.7391	no
S100A9	no	***.0003***	no
SCF	no	.1160	no
SCGF	no	***.0168***	no
TGF-alpha	no	***.0020***	no
TRAIL	yes	***.0017***	no
CCL7	n.a.	n.a.	n.a.
IL-34	n.a.	n.a.	n.a.
LIGHT	no	<.0001	no

PCS⁺, patients with Post-COVID syndrome (PCS); PCS⁻, patients without PCS; ANOVA, analysis of variance; KWT, Kruskal-Wallis test; 1st, first analysis; 2nd, second anlysis; n.a., not applicable. p < 0.05 shows statistical significance.

B CTRL

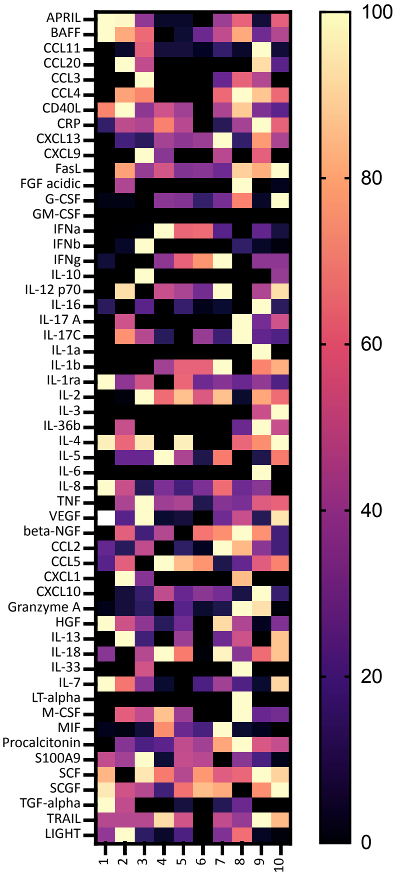

C PCS⁻ T1

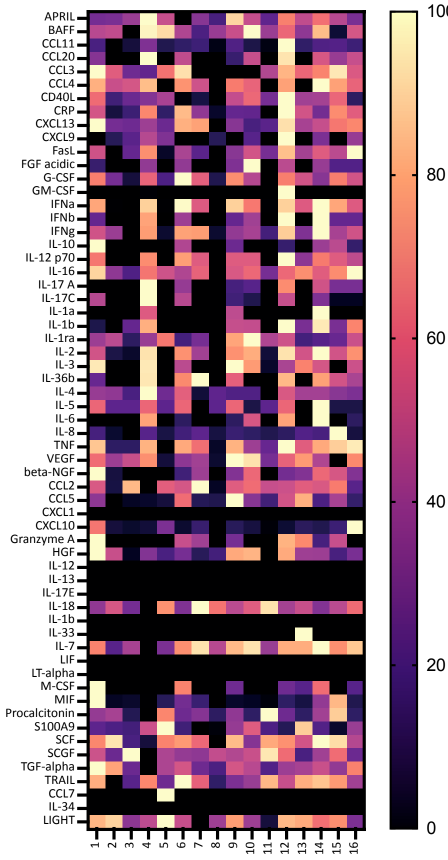

D PCS⁻ T2

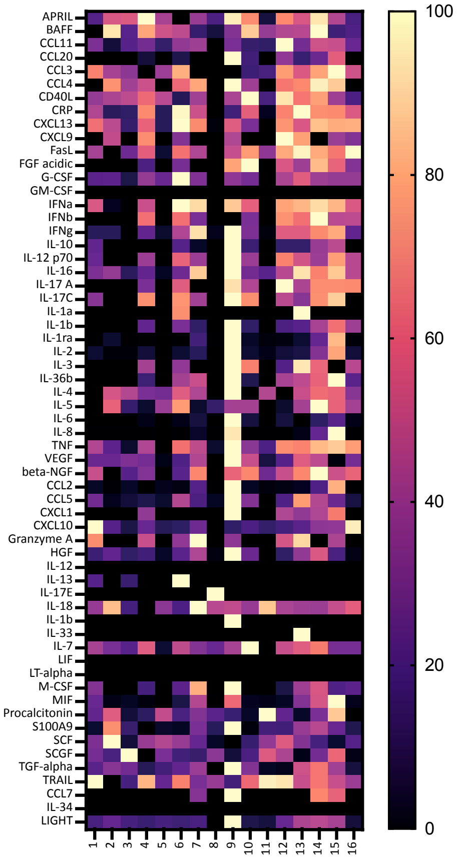

E PCS⁺ T1

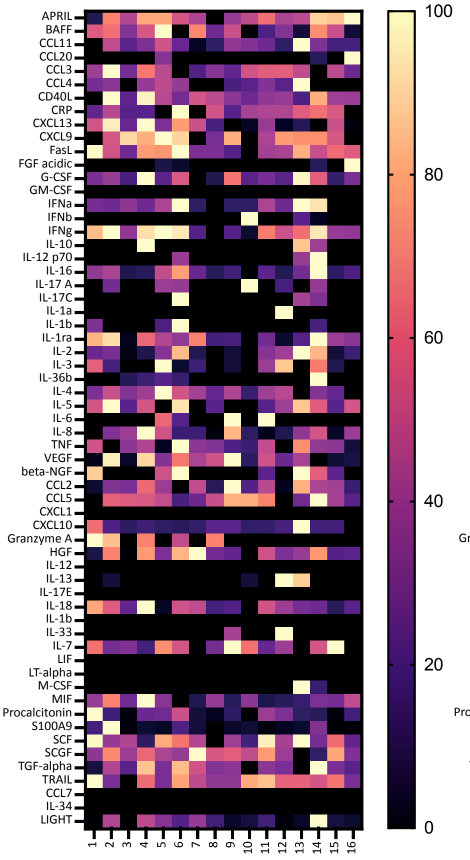

F PCS⁺ T2

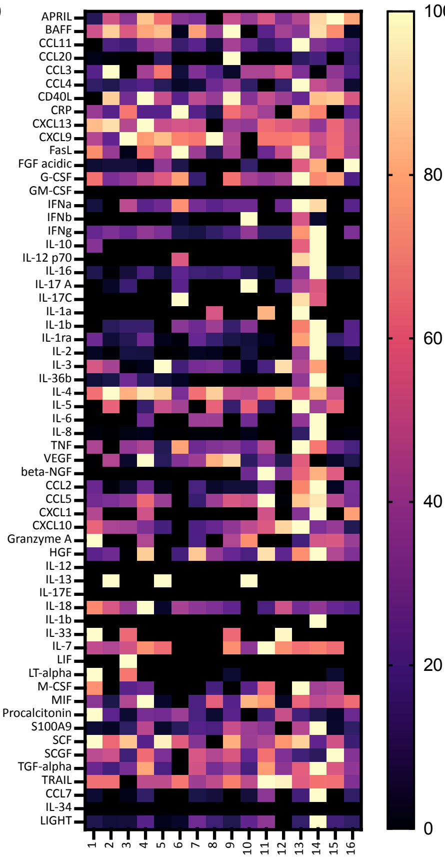

Supplementary Figure 1 Multiplex Luminex cytokine analysis.
A Detailed statistical anlysis of 61 cytokines or chemokines, respectively. **B-F** Heatmap of individual normalized cytokine ratios (%) in CTRL, PCS⁻ and PCS⁺ at T1 and T2, respectively.

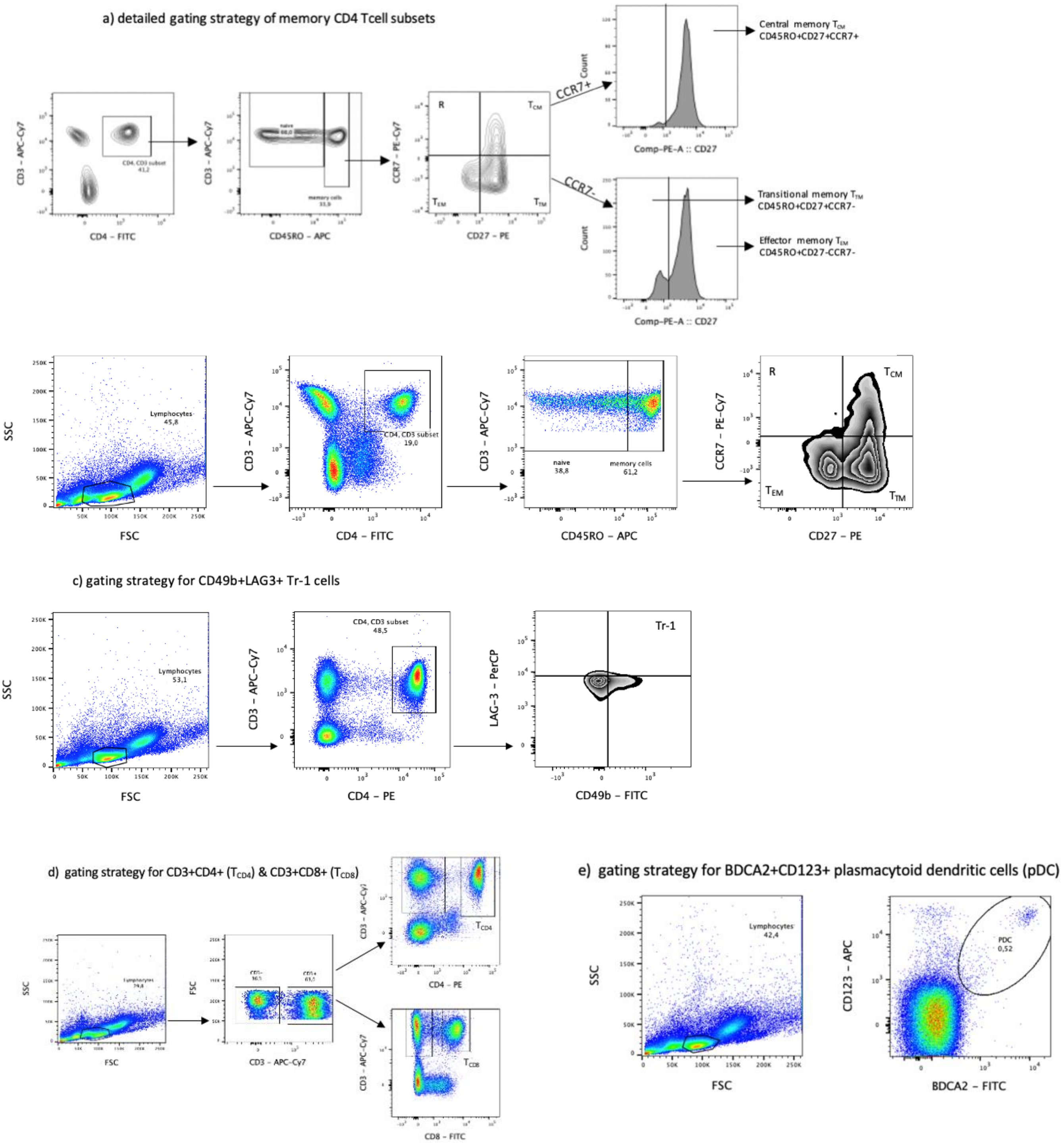

antigen	fluorochrome	clone	isotype	dilution	manufacturer
CD3	APC-H7	SK 7	mouse	1:40	BD
CD4	FITC	RPA-T4	mouse	1:40	BD
CD4	PE	M-T 466	mouse	1:20	Miltenyi Biotec
CD45RO	APC	REA 611	human	1:50	Miltenyi Biotec
CCR7	PE-CY7	3D12	rat	1:80	BD
CD27	PE	M-T 271	mouse	1:40	BD
PD-1	BV 421	EH12.2H7	mouse	1:20	Biolegend
CD49b	FITC	AK-7	mouse	1:20	BD
LAG-3	PerCP	C9B7W	goat	1:10	RD
CD8	FITC	REA 734	human	1:10	Miltenyi Biotec
CD38	PE-Vio770	IB6	mouse	1:50	Miltenyi Biotec
CD32	APC	FUN-2	mouse	1:10	Sony
HLA-DR	PerCP	AC 122	mouse	1:20	Miltenyi Biotec
BDAC-1	PE	AD5-8E7	mouse	1:50	Miltenyi Biotec
BDAC-2	FITC	AC 144	mouse	1:20	Miltenyi Biotec
CD123	APC	AC 145	mouse	1:20	Miltenyi Biotec
CD11c	PE-Vio770	BEA 618	human	1:50	Miltenyi Biotec

Supplementary figure 2. Flow cytometric gating strategy and detailed information on the antibodies and dilutions used.

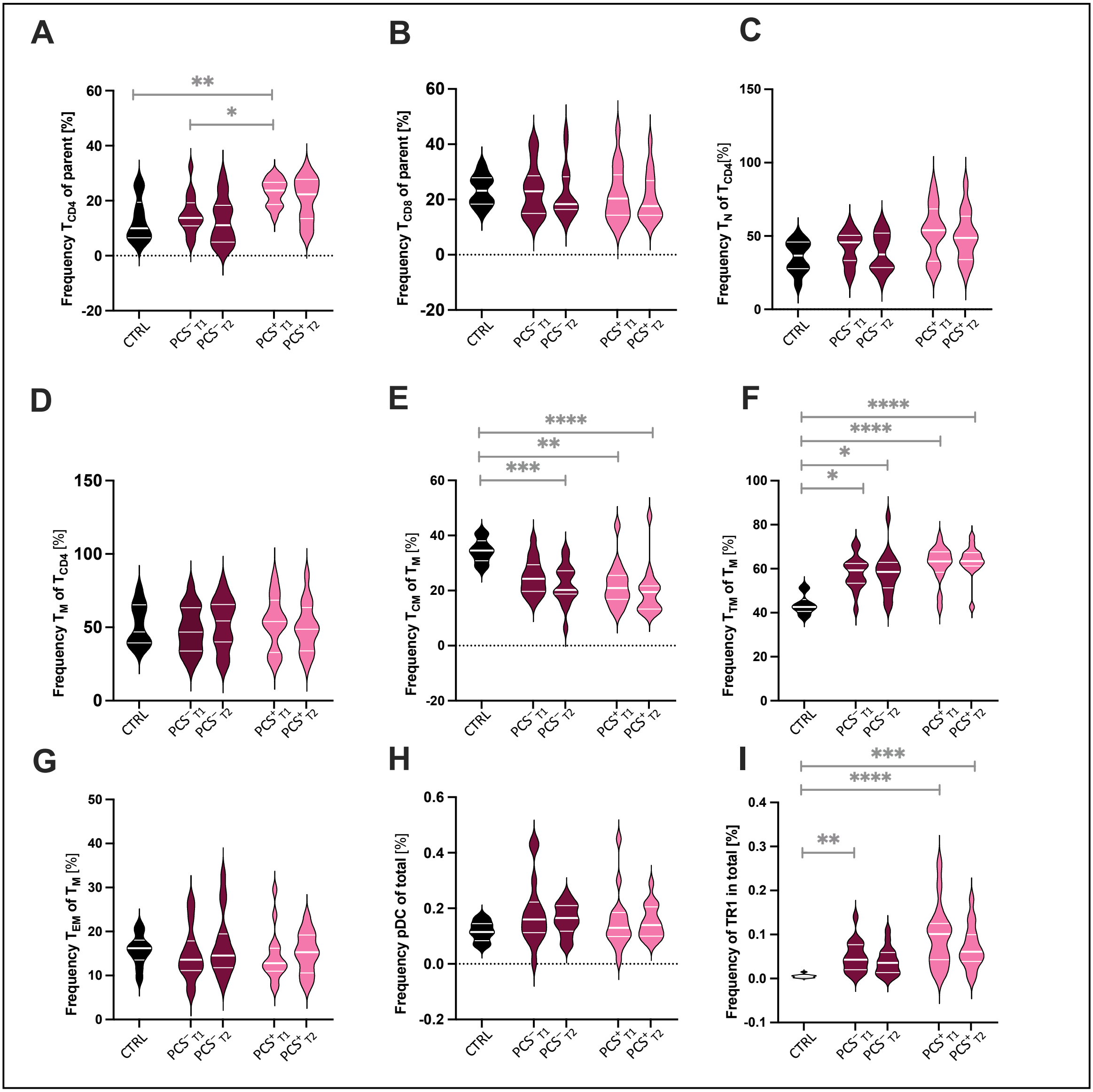

Supplementary figure 3. Distinct cell frequencies by cohort. Frequencies [%] of **A** CD4 positive T-cells (T_{CD4}) of parent **B** CD8 positive T-cells (T_{CD8}) of parent **C** antigen-naive T-cells (T_N) **D** memory T-cells (T_M) of total [%] **E** central memory T-cells (T_{CM}) of T_M **F** transitional memory T-cells (T_{TM}) of T_M **G** effector memory T-cells (T_{EM}) of T_M **H** plasmacytoid dendritis cells (pDC) of total **I** type 1 regulatory T-cells (TR1) of total.

Data information: For statistical analysis, Kruskal-Wallis tests with Dunn's multiple comparisons were used. Here, individual Dunn's multiple comparisons are shown. P-values < 0.05 were considered as statistically significant: * $p \leq 0.05$, ** $p \leq 0.01$, *** $p \leq 0.001$, **** $p \leq 0.0001$, and ns, not significant. PCS^+, convalescents with Post-COVID syndrome (PCS); PCS^-, convalescents without PCS; CTRL, healthy controls, T1, first visit; T2, second visit.

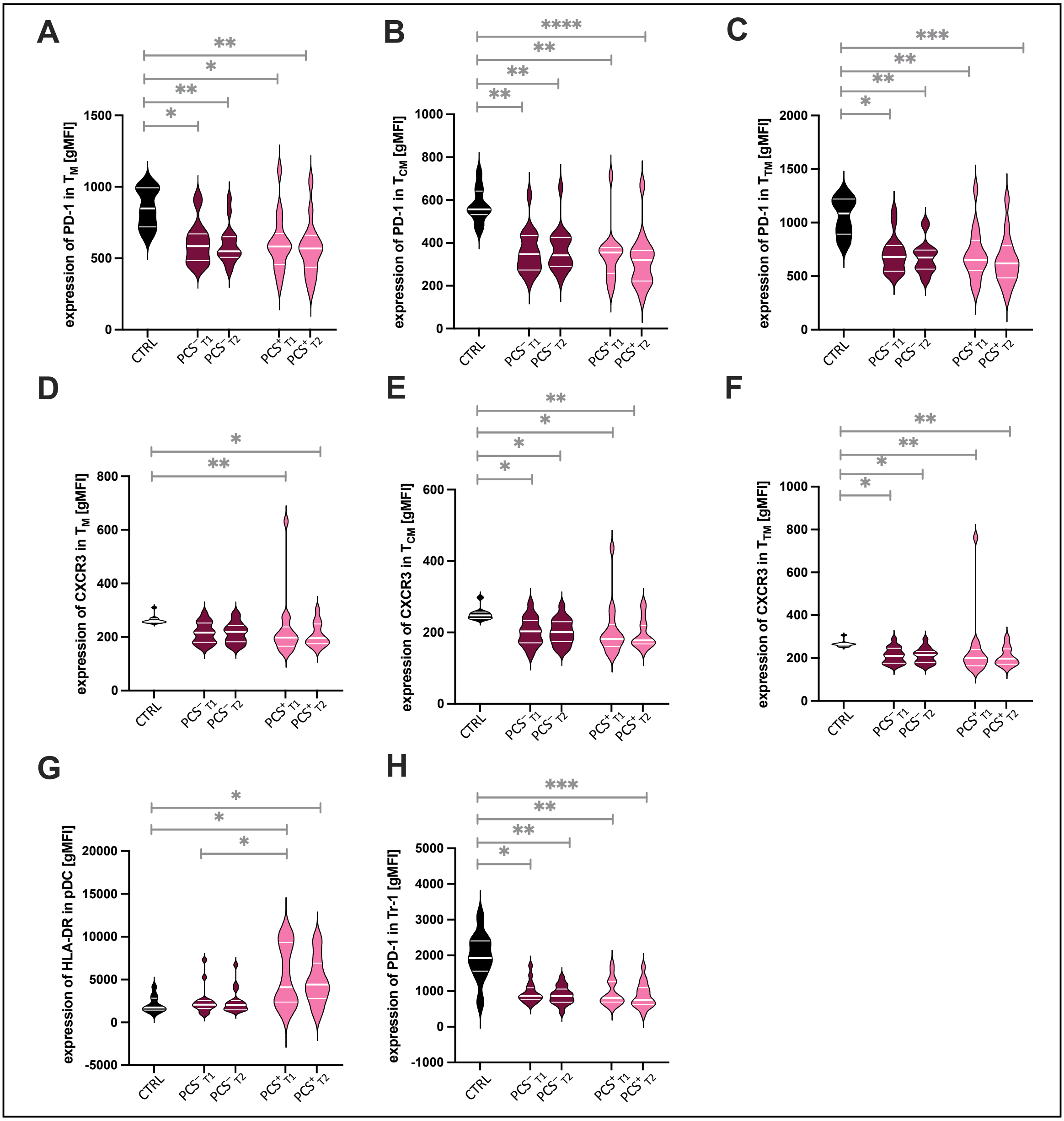

Supplementary figure 4. Surface marker expression by cohort. PD-1 expression [gMFI] on **A** CD4 positive memory T-cells (T_M) **B** $CD4^+$ central memory T-cells (T_{CM}) **C** $CD4^+$ transitional memory T-cells (T_{TM}). CXCR3 expression [gMFI] on **D** T_M **E** T_{CM} **F** T_{TM} **G** human leukocyte antigen DR (HLA-DR) expression [gMFI] on plasmacytoid dendritic cells (pDC). PD-1 expression [gMFI] on **H** type 1 regulatory T-cells (TR1).

Data information: For statistical analysis, Kruskal-Wallis tests with Dunn's multiple comparisons were used. Here, individual Dunn's multiple comparisons are shown. P-values < 0.05 were considered as statistically significant: * p ≤ 0.05, ** p ≤ 0.01, *** p ≤ 0.001, **** p ≤ 0.0001, and ns, not significant. PCS^+, convalescents with Post-COVID syndrome (PCS); PCS^-, convalescents without PCS; CTRL, healthy controls, T1, first visit; T2, second visit; gMFI, geometric mean fluorescence intensity.

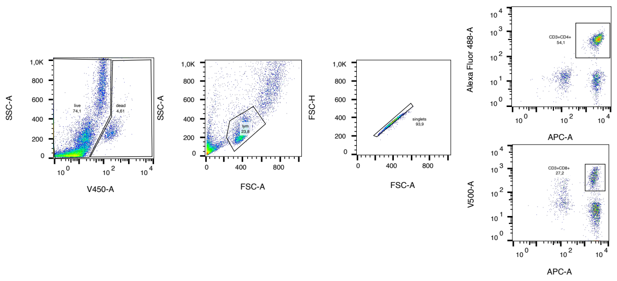

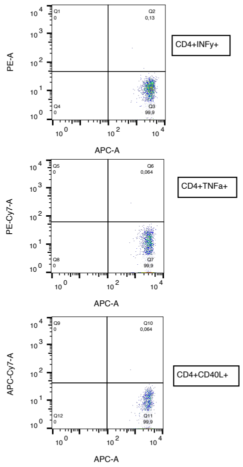

Supplementary figure 5 Detailed FACS gating scheme for T cell assays.

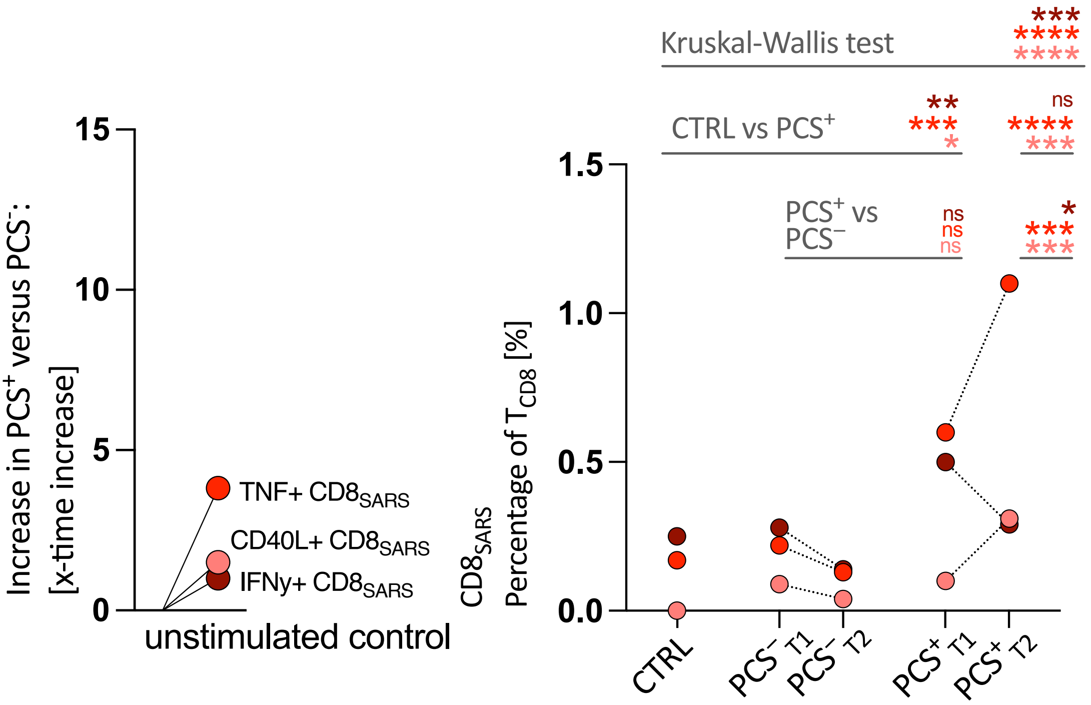

Supplementary figure 6 Detection of distinct SARS-CoV-2 reactive CD8^{+} ($CD8_{SARS}$) T-cells.

Data information: For statistical analysis, Kruskal-Wallis tests with Dunn's multiple comparisons were used. P-values < 0.05 were considered as statistically significant: * p ≤ 0.05, ** p ≤ 0.01, *** p ≤ 0.001, **** p ≤ 0.0001, and ns, not significant. PCS^{+}, convalescents with Post-COVID syndrome (PCS); PCS^{-}, convalescents without PCS; CTRL, healthy controls, T1, first visit; T2, second visit; ns, not significant.
